# Supplementary figures and images for: Multistep loading of a DNA sliding clamp onto DNA by replication factor C
Source: eLife. 2022 Aug 8;11:e78253. doi: 10.7554/eLife.78253 (PMC9359705; doi:10.7554/eLife.78253)

# Figure S1 - source data

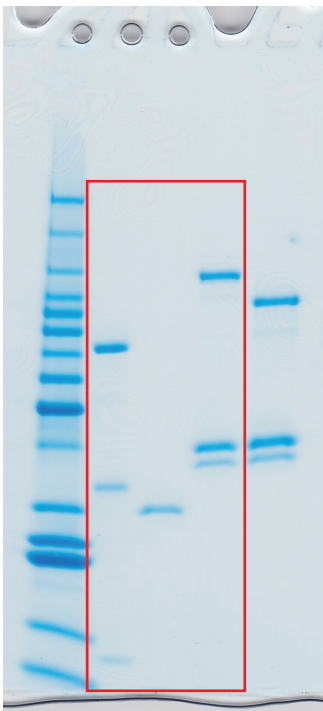

Figure S1A

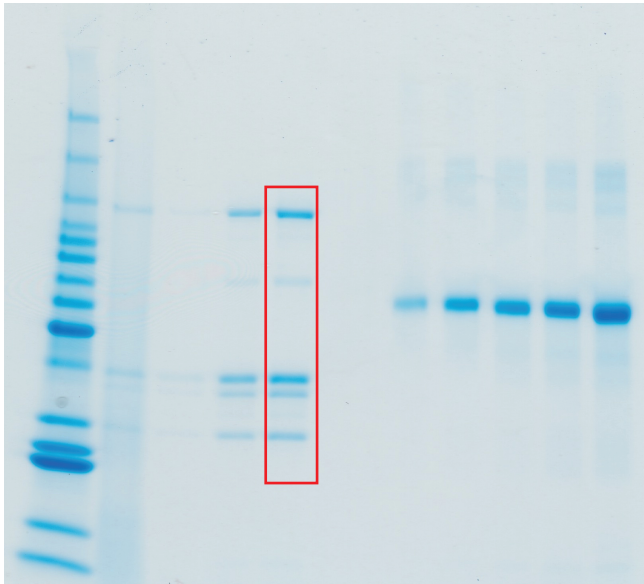

Figure S1E

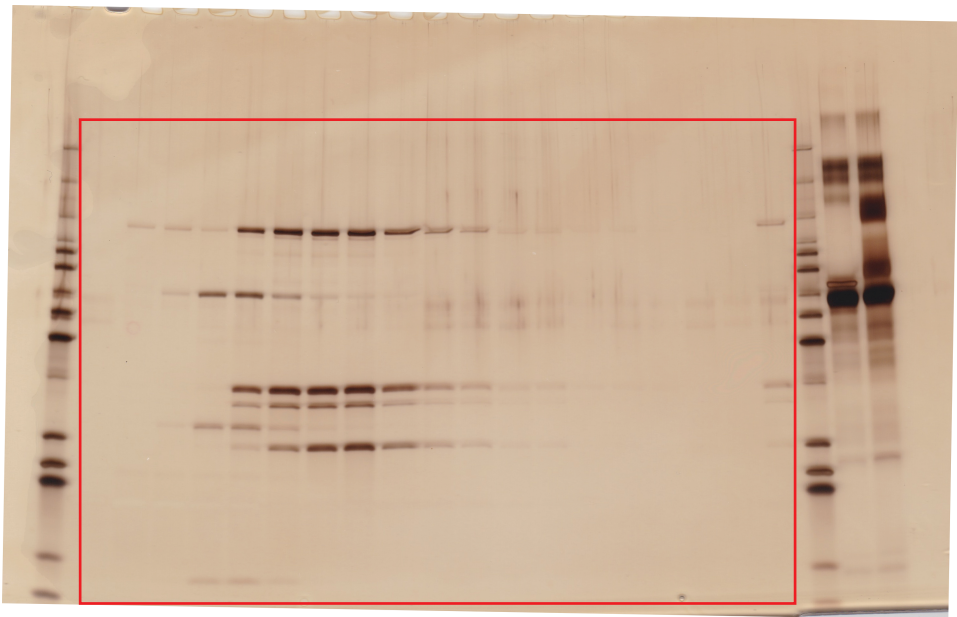

Figure S1D

Supplement: Figure 1—figure supplement 1—source data 1. [file elife-78253-fig1-figsupp1-data1.pdf]

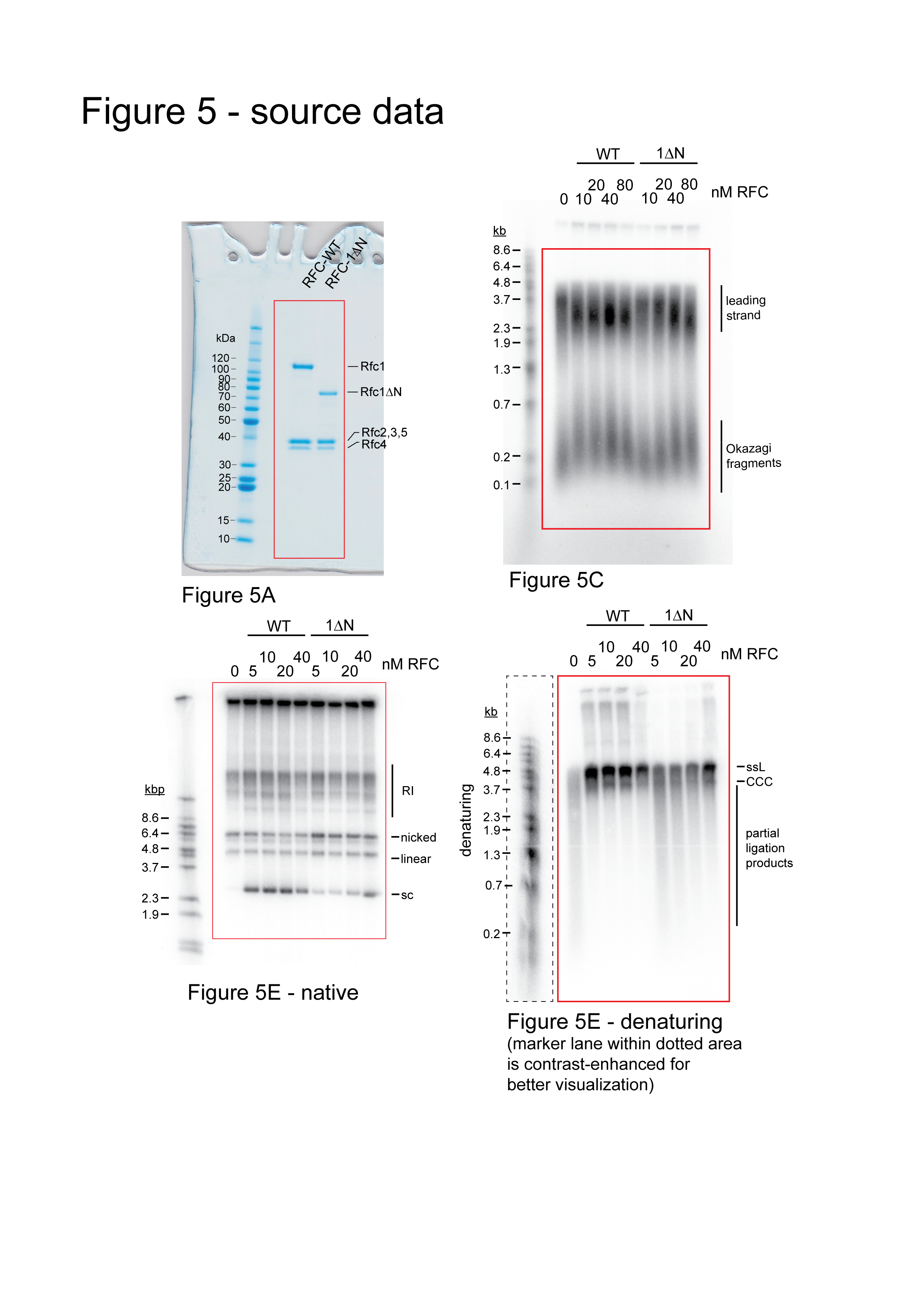

Supplement: Source data 1. [file elife-78253-data1.zip › Sourcedata/Figure 5-source data 1-01.png]

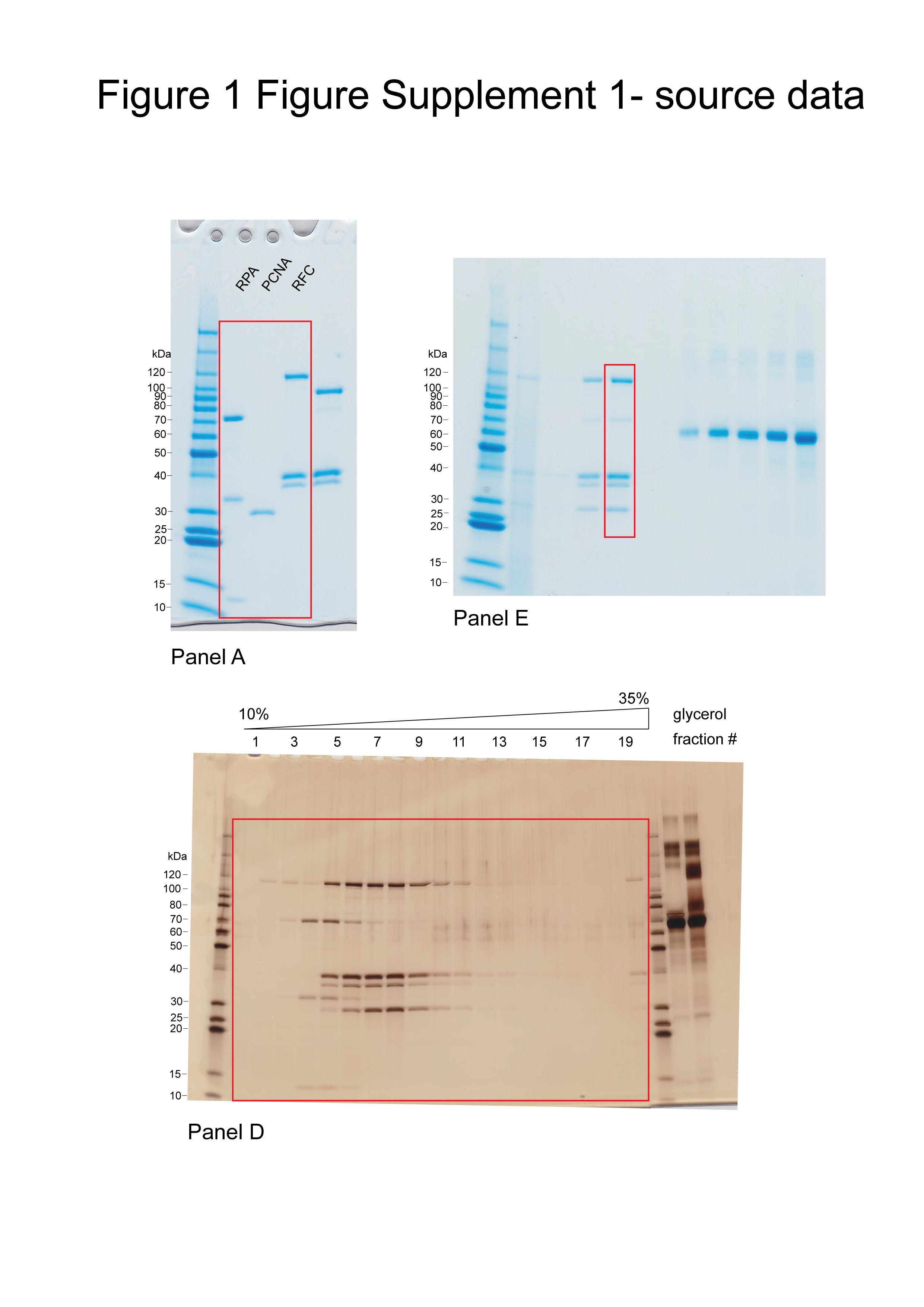

Supplement: Source data 1. [file elife-78253-data1.zip › Sourcedata/Figure1-Source Data 1.png]

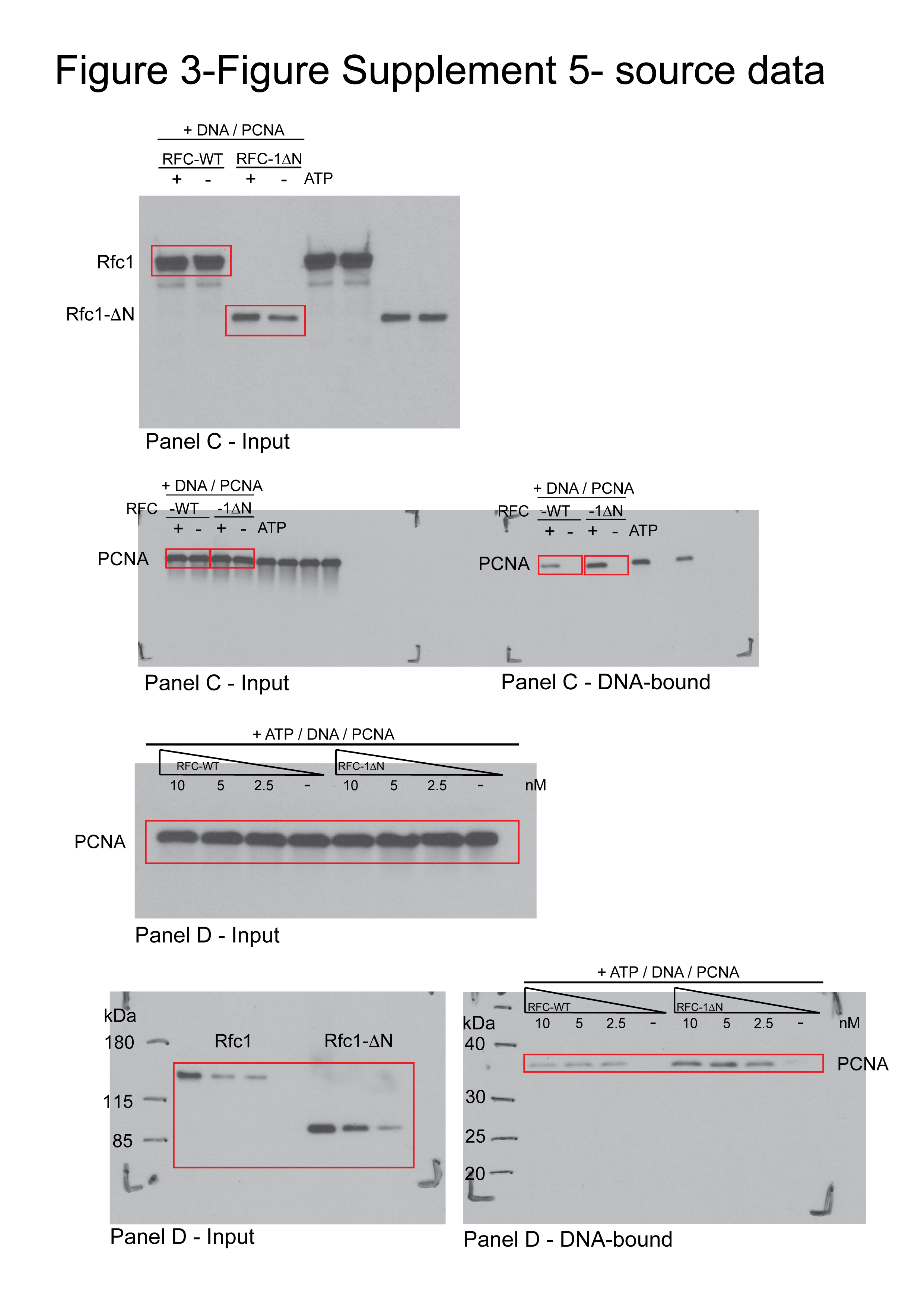

Supplement: Source data 1. [file elife-78253-data1.zip › Sourcedata/Figure 3-source data 1-01.png]
