# Supplementary material for: Multistep loading of a DNA sliding clamp onto DNA by replication factor C
Source: eLife. 2022 Aug 8;11:e78253. doi: 10.7554/eLife.78253 (PMC9359705; doi:10.7554/eLife.78253)
Supplement: Figure 3—figure supplement 5—source data 1. [file elife-78253-fig3-figsupp5-data1.pdf]

Figure 5 - source data

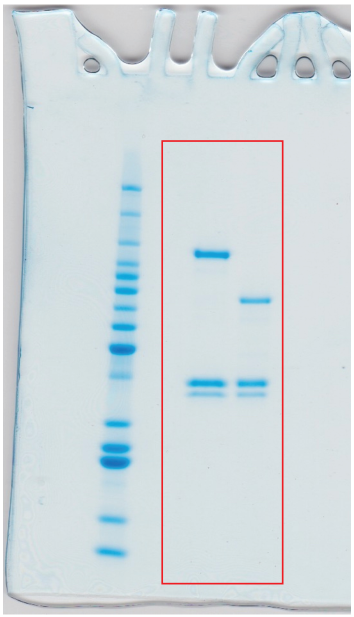

Figure 5A

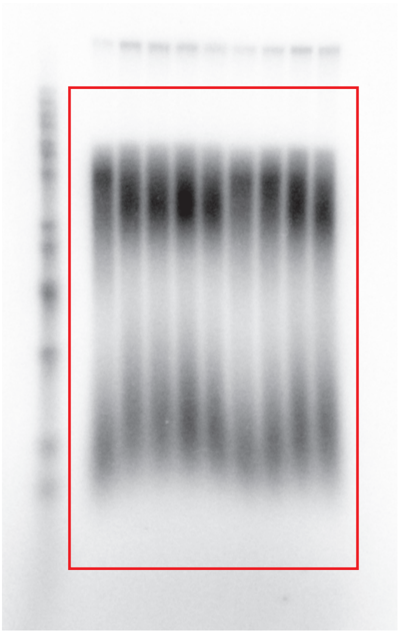

Figure 5C

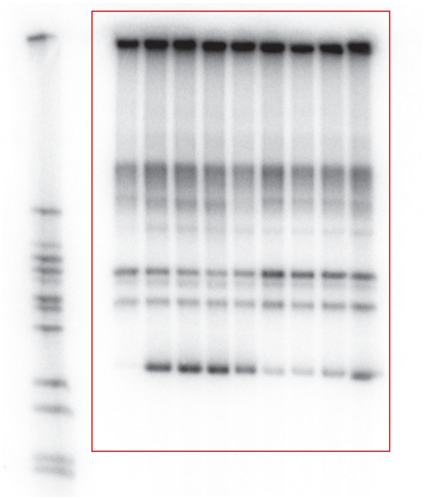

Figure 5E - native

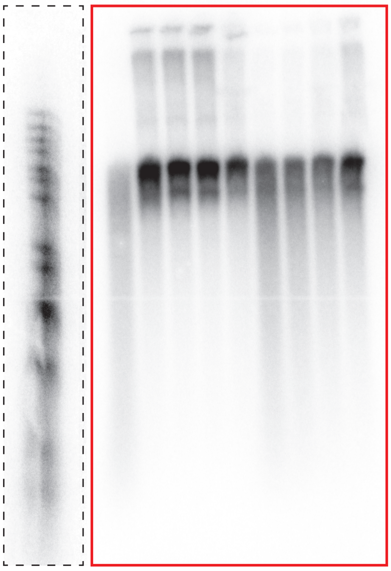

Figure 5E - denaturing
(marker lane within dotted area is contrast-enhanced for better visualization)
