# Supplementary material for: Multistep loading of a DNA sliding clamp onto DNA by replication factor C
Source: eLife. 2022 Aug 8;11:e78253. doi: 10.7554/eLife.78253 (PMC9359705; doi:10.7554/eLife.78253)
Supplement: Figure 5—source data 1. [file elife-78253-fig5-data1.pdf]

Figure S10 - source data

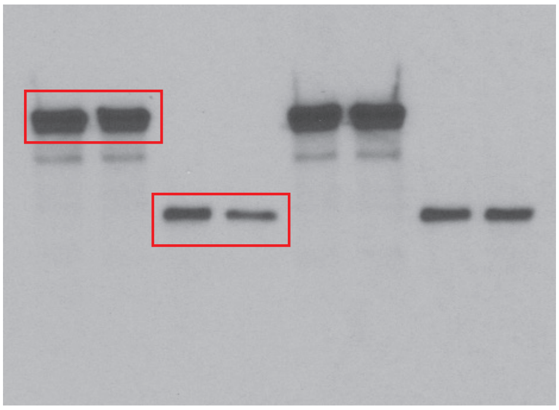

Figure S10C - Input

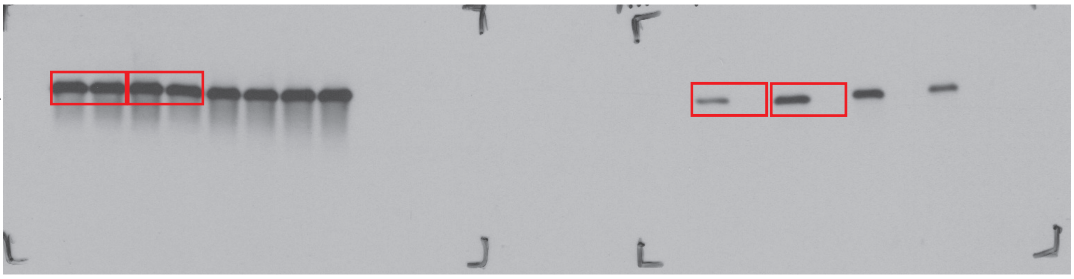

Figure S10C - Input

Figure S10C - DNA-bound

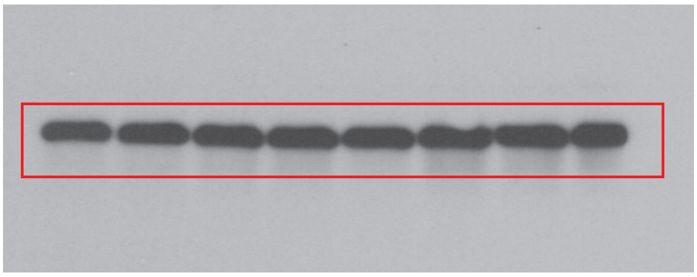

Figure S10D - Input

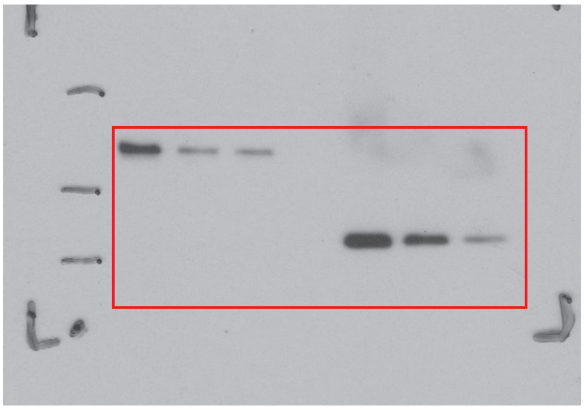

Figure S10D - Input

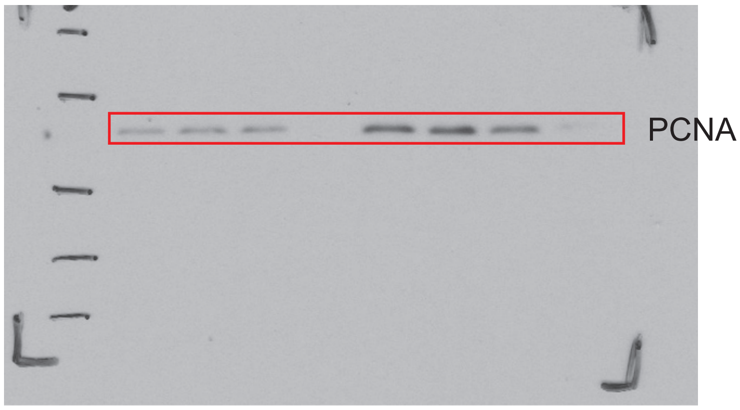

Figure S10D - DNA-bound
